# Subcritical Water Extraction and Hydrolysis of Cod (*Gadus morhua*) Frames to Produce Bioactive Protein Extracts

**DOI:** 10.3390/foods10061222

**Published:** 2021-05-28

**Authors:** Rodrigo Melgosa, Marta Marques, Alexandre Paiva, Ana Bernardo, Naiara Fernández, Isabel Sá-Nogueira, Pedro Simões

**Affiliations:** 1 LAQV-REQUIMTE—Associated Laboratory for Green Chemistry (LAQV) of the Network of Chemistry and Technology (REQUIMTE), Departamento de Química, Faculdade de Ciências e Tecnologia, Universidade Nova de Lisboa, 2829-516 Caparica, Portugal; r.melgosa@fct.unl.pt (R.M.); martadiasmarques@gmail.com (M.M.); abp08838@fct.unl.pt (A.P.); 2 IBET-Instituto de Biologia Experimental e Tecnológica, Food & Health Division, 2781-901 Oeiras, Portugal; ana.bernardo@ibet.pt (A.B.); naiara.fernandez@ibet.pt (N.F.); 3 UCIBIO-REQUIMTE, Applied Molecular Biosciences Unit (UCIBIO) of the Network of Chemistry and Technology (REQUIMTE), Departamento de Ciências da Vida, Faculdade de Ciências e Tecnologia, Universidade Nova de Lisboa, 2829-516 Caparica, Portugal; isn@fct.unl.pt

**Keywords:** cod frames, subcritical water, fish protein hydrolysate, antioxidant activity, antiproliferative activity

## Abstract

The valorization of Atlantic cod (*Gadus morhua*) frames from a filleting industry was investigated using subcritical water extraction and hydrolysis (SBW) at different temperatures (90, 140, 190 and 250 °C) and 100 bar to obtain extracts rich in proteins, peptides and amino acids. Up to 57.7 g of extract per 100 g of codfish frames were obtained, with nearly total recovery of the protein fraction. At each temperature, protein extracts of decreasing molecular weight were obtained, according to SEC-GPC results. Most of the protein present in the raw material and extracts was collagen and collagen fragments, as suggested by the amino acid profile. Codfish SBW extracts did not show cytotoxicity in the range of concentrations tested and the protein extract obtained at the lowest temperature (90 °C) showed the highest anti-inflammatory potential in human intestinal epithelium cell model. The mineralized residue left after SBW treatment of cod frames was identified as practically pure, crystalline, hydroxyapatite, that may find applications in biomedical field and hard-tissue engineering. This study shows the possible valorization of cod frames using green extraction methods such as SBW process to obtain protein extracts for food and nutraceutical applications.

## 1. Introduction

Atlantic codfish (*Gadus morhua*) is among the most important commercial fishes worldwide and the most consumed fish in Portugal. According to recent statistics, Portugal consumes around 70,000 tons per year [1]. This produces large quantities of by-products, mostly skin and bones, which might cause environmental problems when disposed of and should be adequately valorized.

Codfish frames (backbone with residual meat) are regarded as rich in minerals, such as calcium and phosphorus, and collagen proteins, although some carbohydrate and lipids are also found [2]. The bone tissue is mainly built up of an organic extra cellular matrix covered with hydroxyapatite (HAp), a calcium phosphate with chemical formula Ca_10_(PO_4_)_6_(OH)_2_. Both collagen and HAp can be used in a broad range of economically interesting applications as food ingredients, nutraceutics, or cosmetics [3]. Hence, there is a great potential for the conversion of cod frames into different bioproducts with high market value.

Collagen has a wide range of applications as emulsifier, foaming and gelling agent in the food, pharmaceutical, cosmetic, and photographic industries, among others. The most common sources are mainly from porcine or bovine origin. However, fish collagen is less restricted due to religious concerns and transmissible diseases, such as bovine spongiform encephalopathy (BSE) and food and mouth disease (FMD) [4]; thus, it has gained interest in the last years. Fish collagen can be also hydrolyzed to increase its digestibility in nutraceutical preparations and obtain active peptides with biologically active properties (antioxidative, antihypertensive and antimicrobial properties) [5]. Conventional methods for collagen extraction and hydrolysis use thermal treatments and strong acids or alkalis to increase collagen solubility, followed by salt precipitation to allow isolation. Loss of functional and nutritional properties due to the degradation of active molecules with the extreme temperatures and pHs involved, restrains the field of application of these hydrolysates to animal feed and fertilizers, where quality standards are lower [6]. Diluted organic acids such as acetic or citric acid have emerged as alternative extraction solvents, at the cost of increased processing times. Different authors report complex extraction methods consisting of pretreatment, extraction, and purification steps during several days to obtain relatively low yields [7], which is not affordable at industrial scale. Simultaneous or subsequent hydrolysis with proteases has been also introduced to obtain collagen fragments with increased solubility and potential biological properties. Jafarpour et al. [8] have recently investigated the valorization of cod frames by means of enzymatic extraction and hydrolysis. Other authors have proposed the intensification of the process through extrusion methods [7], ultrasound assisted extraction (UAE) [9], or the application of pulsed electric fields [10].

Calcium phosphates such as hydroxyapatite (HAp) present very high value due to their properties and technological applications, being well known for their use as bioceramics [11]. HAp is probably the most important calcium phosphate due to its high biocompatibility, and for this reason it is widely used in the medical field for hard tissue engineering [12]. Other applications of HAp include soil remediation [13], support for nanometallic catalysts [14], and development of fire-proof materials [15]. Most of the HAp and other calcium phosphates are currently produced by chemical synthesis, using different methods [11]. However, natural by-products and biowastes have been also investigated as a source for these materials, with economic and environmental advantages [11,16]. Bovine bones are extensively used to obtain HAp bioceramics [16], although other biowastes such as pig teeth and bones [17,18], chicken bones [19], eggshells [20], fish bones [21,22], and seafood shells [23] have been investigated as potential HAp sources. Different methods for HAp production from biowastes have been explored, such as hydrothermal conversion of calcified materials with phosphorus precursors [16], enzymatic hydrolysis [24], extraction with ionic liquids [25], and the most common thermal treatment at temperatures of 600–1200 °C. The thermal decomposition method is simpler and cost-effective and has reached commercial status [11]. However, collagen proteins and other valuable compounds that might be present in the raw material are destroyed. Hence, previous extraction steps are necessary for an integral valorization of bone residues.

Subcritical water hydrolysis (SBW) represents a green alternative to both protein extraction and hydrolysis processes and HAp production through conventional chemical synthesis or thermal decomposition. SBW uses water at 100–274 °C and pressure above saturation value but less than critical value. At these conditions, the dielectric constant of water decreases with increasing temperature due to hydrogen bond dissociation, allowing water to act as an effective solvent for moderately polar to non-polar substances. Additionally, the ionic product of water (K_W_) increases with temperature, being three orders of magnitude higher than that at ambient conditions. This leads to the increase of hydronium and hydroxide ions concentrations and allows water to act as an acid or base catalyst, facilitating the hydrolysis of lignocellulosic polymers and proteins to smaller oligomers and peptides, without the use of additional catalysts [26,27].

SBW has been widely investigated as a green method for biomass conversion, hemicellulose fractionation and extraction of phenolics [28]. However, SBW can be also applied to proteinaceous materials such as fish and fish wastes, obtaining amino acids and small peptides with interesting physicochemical and bioactive properties. Yoshida et al. [29] reported the SBW of fish meat from horse mackerel in a batch reactor, sequentially producing amino acids, organic acids, and fish oil when increasing temperature from 200 to 400 °C. Other authors have also investigated the SBW of fish waste from different origins, such as white croaker entrails [30,31], bonito [31], squid viscera [32,33], or mackerel meat [34], bones and skin [35,36], among others. Ueno et al. [37] investigated the production of antihypertensive peptides through continuous SBW at 160–200 °C and 20 bar, using cold-water fish gelatin as a fish waste model. Cho et al. [38] studied the production of shrimp (*Penaeus japonicus*) hydrolysates through a batch SBW process at 100–200 °C and evaluated their physicochemical and biofunctional properties, finding strong free-radical scavenging and antioxidant activities that increased with temperature up to 200 °C. Ahmed and Chun [39] compared the hydrolysates obtained from tuna skin and from previously isolated tuna skin collagen through SBW process at 150–300 °C and 50–100 bar. Maximum antioxidant and antimicrobial activities were found at 280 °C and 80 bar for both materials, although hydrolysates from previously isolated collagen showed better results. Asaduzzaman et al. [35] reported the production of bioactive low-molecular-weight peptides from mackerel (*Scomber japonicus*) bone and skin, through a combination of pepsin assisted extraction of collagen and subsequent SBW of the extracts. SBW process was able to increase the antioxidant activity of the extracts, compared to the initially isolated collagen. In a previous work [40], we have recently applied a sequential supercritical fluid extraction and SBW process to the fractionation of sardine (*Sardina pilchardus*) waste into a fish oil rich in omega-3 polyunsaturated fatty acids, and a fish protein hydrolysate fraction rich in peptides and amino acids. Bioactive properties of the hydrolysates were affected by the temperature. The highest antioxidant activity and in vitro antiproliferative effect were found in the extracts obtained at the highest temperature studied, of 250 °C. In all these works, the molecular profile of the hydrolysates showed that low-molecular-weight peptides are associated with the biofunctional activity of the extracts.

Other authors have applied the SBW process to completely remove protein and organic compounds from bone materials, focusing on the production of HAp from the deproteinized residue. Barakat et al. [41] obtained pure HAp nanoflakes from bovine bones by SBW treatment at 275 °C and holding time 1 h, although less crystallinity was obtained with SBW method compared to conventional direct heating treatment at 750–1200 °C. SBW was also used for the deproteinization of shrimp cephalotorax wastes, obtaining highly pure calcareous chitin and promoting the formation of HAp nanocrystals with needle and flake shapes [42].

In this work, the extraction and hydrolysis of protein from cod frames and the simultaneous isolation of HAp using subcritical water have been investigated. To date, SBW process has been applied to the extraction and hydrolysis of collagen from different fish and fish wastes [29,30,31,32,33,34,35,36,37,38,39,40]. However, the production of HAp from these sources is scarcely reported and no mention has been found in the literature about the simultaneous collagen and HAp isolation from bone materials. Thus, the aim of this study was to isolate the main components of the cod frames and characterize their potential application as functional ingredients, to promote a more effective use of the waste generated during codfish processing.

## 2. Materials and Methods

### 2.1. Materials

Atlantic codfish (*Gadus morhua*) frames resulting from the industrial fish filleting process and consisting of the fish backbone and adhered muscle were provided by Pascoal and Filhos S.A. (Gafanha da Nazaré, Portugal). Each batch of fish waste was received fresh, immediately lyophilized, and kept at −20 °C. Prior to experiments, samples were grinded (IKA Tube-Mill Control, IKA^®^-Werke GmbH & Co. KG, Staufen, Germany) and manually crushed to a fine powder (ca. 5 mm). During this process, bones from different fish and from different parts of the same fish were mixed, to ensure that the samples were as homogenous as possible.

Lowry reagent, 2N Folin-Ciocalteu’s reagent, Bovine Serum Albumin (BSA) standard, phenol (99%), d(+)-glucose monohydrate, Bradford reagent and Lipopolysacccharides from *Escherichia coli* O55:B5 (LPS) were obtained from Sigma-Aldrich (Saint Louis, MO, USA). Human colorectal adenocarcinoma cell lines, Caco-2 and HT-29 were obtained from Deutsche Sammlung von Mikroorganismen und Zellkulturen (Braunschweig, Germany) and American Type Culture Collection (Manassas, VA, USA), respectively. Cell culture media (Dulbecco’s Modified Eagle Medium, DMEM, and Roswell Park Memorial Institute, RPMI) and supplements (heat inactivated Fetal Bovine Serum, FBS, and non-essential amino acids, NEAA) were purchased from Invitrogen (Gibco, Invitrogen Corporation, Paisley, UK). CellTiter 96 Aqueous One Solution Cell Proliferation Assay (MTS) was obtained from Promega (Madison, WI, USA). Human IL-8 (CXCL8) Mini TMB ELISA Development Kit and TNF-α human recombinant were obtained from Peprotech (London, UK). Human IL-1β was obtained from Sino Biological (Düsseldorf, Germany). All other reagents and solvents used in the present study were of analytical grade and purchased from available suppliers. 

### 2.2. Subcritical Water Extraction and Hydrolysis (SBW)

The subcritical water hydrolysis (SBW) of codfish frames was performed in a lab-scale apparatus described in previous publications [27]. Briefly, it consists of a distilled water reservoir, a Knauer 1800 high-pressure pump (Knauer Wissenschaftliche Geräte GmbH, Berlin, Germany), and a high-pressure reactor (High Pressure Equipment Co., Erie, PA, USA) with 510 mm length, 50 mm O.D., and 26 mm I.D., placed in a Nabertherm TR 240 electrical oven (Nabertherm GmbH, Lilienthal, Germany). Pressure of the system was controlled by a Tescom back-pressure regulator (BPR). Water mass flow and total mass of water fed to the system was recorded by means of a Rheonik RHM 007 mass flow meter (Rheonik Messtechnik GmbH, Odelzhausen, Germany).

In a typical experiment, 60 g of grinded codfish frames were loaded into the reactor, which was subsequently placed in the electrical oven and connected to the high-pressure circuit. Then, the pump was turned on at a flow rate of 10 mL/min and the BPR was set at 100 bar to pressurize the system. When pressure reached 100 bar, the BPR opened and extract collection started, initiating the experiment. The preheating wires and the electrical oven were turned on at that point, and outlet temperature was increased from ambient conditions to 250 °C. Samples were continuously collected in different flasks at specific target temperatures (90, 140, 190 and 250 °C), which were maintained constant for 30 min. For each sample, 25 mL were taken in triplicate, lyophilized, and weighed to calculate the extraction yield. The rest were frozen and kept at −18 °C for further analysis. The residue that remained in the reactor after SBW experiments was washed with water, dried at 105 °C overnight, weighed and stored at 4 °C for further analysis.

### 2.3. Proximate Composition

Moisture was determined in a Kern DAB 100-3 thermogravimetric balance (Kern & Sohn GmbH, Balingen, Germany) at 105 °C. Ash content was gravimetrically analyzed through calcination in a muffle at 550 °C during 4 h.

The protein content was determined by elemental analysis of nitrogen in a Flash EA 1112 CHNS analyzer (Thermo Fisher Scientific, Marietta, OH, USA), applying an appropriated nitrogen-to-protein conversion factor (NPCF).

A modified Lowry method [40] was also used to provide a second measurement of the protein content of codfish frames and protein extracts. Protein extraction from codfish frames was performed by alkaline hydrolysis with 4.2 M NaOH at 95 °C during 1 h (S:L = 1:20). Lyophilized protein extracts were redissolved in distilled water at a known concentration. Absorbance at 750 nm was read in a Genesys 50 spectrophotometer (Thermo Fisher Scientific, Marietta, OH, USA) and protein concentration in the samples was calculated based on a calibration curve made with BSA standard at concentrations ranging from 40 to 400 μg/mL. Blank runs were also performed with distilled water instead of the protein sample.

Fat content was measured through the Bligh and Dyer [43] method. Soxhlet extraction with *n*-hexane was also performed in order to estimate the amount of non-polar lipids, following the method described by Pedras et al. [27]. Total sugars were determined by phenol-sulphuric acid colorimetric method, which is described elsewhere [27].

### 2.4. Amino Acid Profile

The amino acid profile of codfish frames and protein extracts obtained after SBW process was analyzed using a Dionex ICS-3000 high-performance liquid chromatography–ion chromatography (HPLC-IC) system (Thermo Fisher Scientific, Marietta, OH, USA). The chromatographic separation was carried out at 30 °C in a Dionex AminoPac PA10 IC column (250 × 4 mm), with a 50 × 4 mm pre-column. Detection was performed in an electrochemical (ED) detector. Solvent flow was 0.8 mL/min and gradient elution was achieved with solvents (S) S1: water, S2: 250 mM NaOH, S3: 1 M sodium acetate, S4: 0.1 M acetic acid. The course of the gradient was as follows: elution time (t) = 0: 76% S1, 24% S2; t = 8 min: 64% S1, 36% S2; t = 18 min: 40% S1, 20% S2, 40% S3; t = 23 min: 14% S1, 16% S2, 70% S3; t = 45 min: 100% S4; t = 47.1 min: 20% S1, 80% S2; t = 49.3 min: 76% S1, 24% S2 (maintained for 15 min). Sample preparation consisted of an alkaline digestion with 4.2 M NaOH for 24 h at 110 °C (S:L = 1:20). After proper neutralization and dilution, 10 µL were injected and HPLC-IC analysis was performed in triplicate. Individual amino acids were identified and quantified using calibration curves constructed with the corresponding standard compounds and using norleucine as internal standard. All compounds were obtained from Sigma-Aldrich (Saint Louis, MO, USA).

Free amino acids present in the SBW extracts were determined following the same analytical procedure but omitting the alkaline digestion step. Amino acid profile of codfish frames was used to calculate a corrected nitrogen-to-protein factor, following the NREL protocols [44]. Additionally, the weight percentage of collagen in the codfish frames and the SBW extracts was estimated from the hydroxyproline content as in Equation (1) [45]:

(1) % collagen=% Hyp×7.46

where % Hyp is the weight percentage of hydroxyproline in each sample. This value was used to monitor the collagen purity of the extracts.

### 2.5. Mineral Profile

To evaluate the mineral composition and the potential presence of heavy metals in the codfish frames, the obtained protein extracts, and the isolated HAp, inductively coupled plasma–atomic emission spectrometry (ICP-AES) analysis was performed using a Horiba Jobin-Yvon Ultima ICP (Horiba Ltd., Kyoto, Japan), equipped with a 40.68 MHz RF generator and a Czerny-Turner monochromator. Argon was used to generate the plasma. To prepare the samples, the mineralized residue from ash determination was dissolved in 6 M nitric acid at a known concentration and subsequently diluted with distilled water.

### 2.6. SDS-PAGE

Sodium dodecyl sulfate–polyacrylamide gel electrophoresis (SDS-PAGE) of the protein extracts obtained after SBW process of codfish frames was conducted by the method of Laemmli [46], with some modifications, to separate the proteins by their molecular weight. The hydrolysates, 100 μg protein of each sample, were mixed with the loading buffer (50 mM Tris-HCl, pH 6.8, 2% (*w*/*v*) SDS (*w*/*v*), 10% glycerol (*v*/*v*), and 0.01% (*w*/*v*) bromophenol blue), and then loaded onto two different polyacrylamide gels: (i) a 7.5% precast gel, and (ii) a 15% resolving gel and 4.5% stacking gel. The electrophoresis was conducted at a constant voltage of 120 V. After electrophoresis, the gel was stained with 0.25% (*w*/*v*) Coomassie Blue R-250 in 8% (*v*/*v*) acetic acid and 46% (*v*/*v*) ethanol for 30 min and subsequently destained with a solution containing 10% (*v*/*v*) acetic acid and 30% (*v*/*v*) ethanol. A molecular-weight protein marker (NZYColour Protein Marker II) (NZYTech, Lda.—Genes and Enzymes, Lisbon, Portugal) was used to estimate the mass of the protein bands.

### 2.7. Size Exclusion–Gel Permeation Chromatography (SEC-GPC)

The extracts obtained after SBW treatment of codfish frames were filtrated through nylon syringe filters (0.22 µm pore diameter, ALWSCI Corporation, Zhejiang, China) and submitted to size exclusion–gel permeation chromatography (SEC-GPC) to estimate their molecular mass distribution. The equipment used was a Knauer Smartline HPLC system. For each sample, 20 µL were injected into a Phenomenex Yarra SEC-2000 column (300 × 7.8 mm i.d.) maintained at a constant temperature of 25 °C, and eluted with 0.1 M phosphate buffer (pH 6.8) including 0.025% sodium azide at a flow rate of 1.0 mL/min. The relative intensity of the eluate was monitored with a UV/Vis detector at 280 nm. The retention times of molecular mass standards (Protein Standard Mix 69385 from Sigma-Aldrich (Saint Louis, MO, USA)) were used to calculate the molecular mass distribution of the SBW extracts according to the retention time of the different peaks present in the chromatogram.

### 2.8. Cell Viability

To evaluate the potential of codfish SBW extracts for nutraceutical applications, cell viability assays were performed with the SBW extracts obtained at different temperatures. Human colorectal adenocarcinoma cell lines, Caco-2, were cultured following previously described methods [47]. Firstly, cytotoxicity effects were assessed through MTS assay using confluent Caco-2 cells as a model of the human intestinal epithelium. Briefly, cells were seeded in 96-well plates at a density of 2 × 10^4^ cells/well and allowed to reach confluence for 7 days. Subsequently, the cells were incubated for 24 h with the extracts diluted in culture medium (DMEM + 0.5% FBS + 1% NEAA). The range of concentrations tested was 0.39–50 mg/mL. Cells incubated with only culture medium were considered as control. After 24 h of treatment, the medium was removed and cell viability was measured using MTS reagent, according to a previously described method [47]. Three independent experiments were performed in a microplate spectrophotometer at 490 nm (EPOCH 2, BioTek Instruments, Winooski, VT, USA). Cell viability was expressed in terms of percentage of viable cells relative to the control and half maximal effective concentration (EC_50_) values were calculated from dose–response curves, as the concentration of sample necessary to decrease cell proliferation by 50%.

### 2.9. Anti-Inflammatory Potential of Hydrolysates

Anti-inflammatory properties of codfish SBW extracts were tested in Caco-2 cell line. Cells were plated at a density of 100,000 cells/cm^2^ and allowed to grow for 21 days, with medium (DMEM supplemented with 1% NEAA, 10% FBS and 1% PS) renewed 3 times a week. On the 21st day cells were ready to be used as a model of inflammation of the intestinal mucosa. To mimic the inflammation, cells were stimulated for 48 h with a cocktail of cytokines (25 ng/mL IL-1β, 50 ng/mL TNF-α and 10 ng/mL LPS) and extracts diluted in medium (DMEM supplemented with 1% NEAA and 0.5% FBS). The extract concentration tested was 25 mg/mL since it did not show any cytotoxic effect. Cells incubated only with medium were considered as negative control and cells incubated with medium and the cocktail of cytokines were considered positive control. After 48 h of treatment the medium was collected and centrifuged at 2000× *g* for 10 min. The supernatant was recovered and stored at −80 °C until the ELISA.

### 2.10. Quantification of IL-8

According to the cocktail of cytokines used, ELISA was performed to detect the presence of IL-8. The assessment of the IL-8 levels in the supernatant were performed using the human IL-8 (CXCL8) Mini TMB ELISA Development Kit (Peprotech, London, UK), according to the protocol provided by the manufacturer. Briefly, one day prior to running the assay, 96-well plates were coated with the capture antibody. Following overnight incubation at room temperature, the plates were washed with wash buffer (PBS (Thermo Fisher Scientific, Marietta, OH, USA) containing 0.05% Tween-20 (Sigma-Aldrich, St. Louis, MO, USA) and then incubated for 1 h at room temperature (RT) with block buffet (1% BSA in PBS) to inhibit nonspecific binding. After washing, 100 mL of standard dilution or extracts was added to each well in triplicate and incubated for 2 h at RT. After washing the plates, 100 mL of detection antibody was added to each well. The plates were then incubated for 2 h at RT, and then washed again, followed by the incubation with 100 mL of streptavidin-horseradish peroxidase (HRP) conjugated for 30 min at RT. Next, after additional washing, 3,3’,5,5’-tetramethylbenzidine (TMB) substrate solution was added and the plates were incubated in the dark at RT for the color development (approximately 20 min). The reaction was stopped by the addition of 100 mL of HCL 1M, and the absorbance was measured at 450 nm with wavelength correction set at 620 nm in a microplate spectrophotometer (EPOCH 2, BioTek Instruments).

The results (mean ± SD of at least three experiments) were expressed in percentage normalized to the positive control.

### 2.11. Hydroxyapatite Isolation and Characterization

The deproteinized residue remaining in the SBW reactor was dried and submitted to different instrumental techniques to determine the presence and purity of HAp. For comparison, the product obtained after direct heating of codfish frames at 550 °C during 5 h, and the residue obtained after alkaline digestion (4.2 M NaOH, 110 °C, 24 h, S:L = 1:20) were analyzed by the same instrumental techniques, Fourier Transform-Infrared (FT-IR) spectroscopy, X-ray diffraction (XRD), and Field Emission Gun Scanning Electron Microscopy (FEG-SEM)as follows.

Spectroscopic characterization of codfish frames, lyophilized protein extracts, and the remaining residue after SBW process was performed by FT-IR spectroscopy. The spectra between 4000 and 400 cm^−1^ of all the samples were recorded as KBr discs, using a Perkin-Elmer Spectrum Two FT-IR spectrometer (Waltham, MA, USA). KBr discs were pressed using 5 mg of the sample mixed with 200 mg of desiccated KBr. The FT-IR spectra of Calcium phosphate hydroxide (HAp) from the National Institute of Standards and Technology (NIST) was used as a reference standard [48]. Data about the phase and crystallinity of the samples from the remaining residue were determined by XRD using a benchtop X-ray Diffractometer Rigaku, model MiniFlex II, with Cu K-α radiation (30 KV/15 mA) (Tokyo, Japan). Measurements were taken at a scan speed of 1°/min and 0.02° steps, between 20 and 55°. The NIST standard for HAp (SRM 2910b [49]) was used to identify the phases and compare the samples. FEG-SEM was performed in a JEOL 7001F equipment (Jeol Ltd., Tokyo, Japan) with Oxford light elements and Energy-Dispersive Detector (EDS). Samples were gold-sputtered in Ar plasma prior to analysis.

### 2.12. Statistical Analysis

SBW experiments were duplicated. Analytical data are expressed as mean ± standard deviation (SD) of triplicates. Significant differences were detected through one-way analysis of variance and Fisher’s LSD test, using Statgraphics software (version 18 for Windows).

GraphPad Prism 6 software was used to calculate the EC50 values of cytotoxicity. Results of potential anti-inflammatory analyses are the averages of at least three independent experiments and are reported as mean % positive control ± SD. Differences between the controls and the experimental concentrations were assessed using *t*-test (*p* < 0.05) using the GraphPad Prims 6 software.

## 3. Results and Discussion

### 3.1. Characterization of Raw Material

The main results obtained in the characterization of the raw material are summarized in Table 1. As expected, protein and ash were the major components of codfish frames. Protein content was analyzed through several methods, obtaining differing results. The Lowry method gave the lowest protein content, with 38.9 ± 2.7 g/100 g. The Lowry method is a widely used colorimetric technique based on the binding of the Folin–Ciocalteu’s reagent with aromatic amino acids. Due to its simplicity and availability, it has been widely used for protein determination in food and food wastes. However, many compounds besides aromatic amino acids can react with the Folin–Ciocalteu’s reagent, leading to strong interferences. In addition, the amino acid sequence of the protein also affects the analysis. In the case of codfish frames, where collagen is the major protein, the low concentration of aromatic amino acids can lead to underestimations of the protein content [50].

Determination of the protein content based on nitrogen analysis assumes that all nitrogen in the sample is protein-bound and all proteins have the same nitrogen content of 16%, leading to the typical NPCF of 6.25. With this method, protein content of codfish frames was estimated to be 57.6 ± 0.7 g/100 g. However, the assumptions made by this method are quite rough as the relative content of nitrogen varies between amino acids and amino acid composition varies between food proteins [51]. In addition, the presence of non-protein nitrogen compounds such as nitrates, ammonia, urea, nucleic acids, free amino acids, chlorophylls, and alkaloids can lead to overestimations of the protein content.

The analysis of the amino acid profile by HPLC-IC permits to estimate the protein content of codfish frames. The total sum of amino acids as determined by this method gives an intermediate value for the protein content of 46.5 ± 0.9 g/100 g. Nonetheless, this method assumes that no free amino acids are present in the raw material and relies on a protein hydrolysis step prior to analysis, which in this work was carried out in 4.2 M NaOH at 110 °C for 24 h. Harsh conditions efficiently hydrolyze most of the peptide bonds, but some amino acids can be further destroyed, leading to an underestimation of the protein content [50].

The amino acid profile allows to calculate a corrected NPCF by considering the actual nitrogen content of each amino acid. Following the National Renewable Energy Laboratory (NREL) protocols [44], the specific NPCF for codfish frames was calculated to be 5.1 ± 0.3. This value is lower than the typical factor of 6.25 and corresponds to a higher average presence of nitrogen in the codfish frames proteins, of 19.6%, which is closer to the average value for collagen containing meat of 18% [52]. Based on this corrected NPCF and the analysis of nitrogen, the protein content of codfish frames was determined to be 47.0 ± 0.9 g/100 g. Though all the protein analysis methods used in this work have their drawbacks, we adopted the last value as the actual protein content for subsequent calculations. This value is close to the one obtained by the sum of amino acids, demonstrating low degradation during the alkaline hydrolysis step.

The protein content of codfish frames as determined by the corrected NPCF is higher than the value reported by Toppe et al. [2]. However, in their case, all possible adhered muscle was removed by further processing the raw material in boiling water, whereas the codfish frames used in this work were not boiled. This might also explain the lower ash content observed in this work compared to them. The low lipids content of codfish frames agrees with the previously reported values [2], which are characteristic of lean fish that store fat in their liver. Carbohydrate content of codfish frames was found to be very low, as expected.

Regarding the relative amino acid composition of codfish frames (Table 1), we can see high contents of glycine, alanine, and proline, as well as hydroxyproline (8.8%) that is almost exclusively found in collagen. The hydroxyproline content in codfish frames protein is higher than that previously reported for *Salmo salar* skin (5.68 g per 100 g of protein) but similar to that for bovine achilles tendon and femur (respectively, 8.15 and 10.79 g per 100 g of protein) [53]. According to Equation (1), collagen content of codfish frames can be estimated from the hydroxyproline content. The value obtained for the codfish frames studied in this work was 30.6 ± 0.8 g of collagen per 100 g of codfish frames, which accounts for roughly 2/3 of the total proteins present in the original material. This value is comparable to those found in codfish skin [54,55].

Mineral profile is similar to that reported by Toppe et al. [2]. Calcium and phosphorous are the major mineral compounds, followed by sodium and magnesium. Minor elements are zinc and iron. The Ca/P ratio, which can be used as an indication of the presence and purity of hydroxyapatite, was 1.801. This value is higher than the stoichiometric 1.667, although it lies within the acceptable ranges for hydroxyapatite [56]. The presence of common heavy metals was also monitored. Most of them were absent or present within legal limits, which opens the utilization of codfish frames derived materials in human food and pharmaceutical applications.

### 3.2. Extraction Yield and Extract Composition

**Figure 1:**
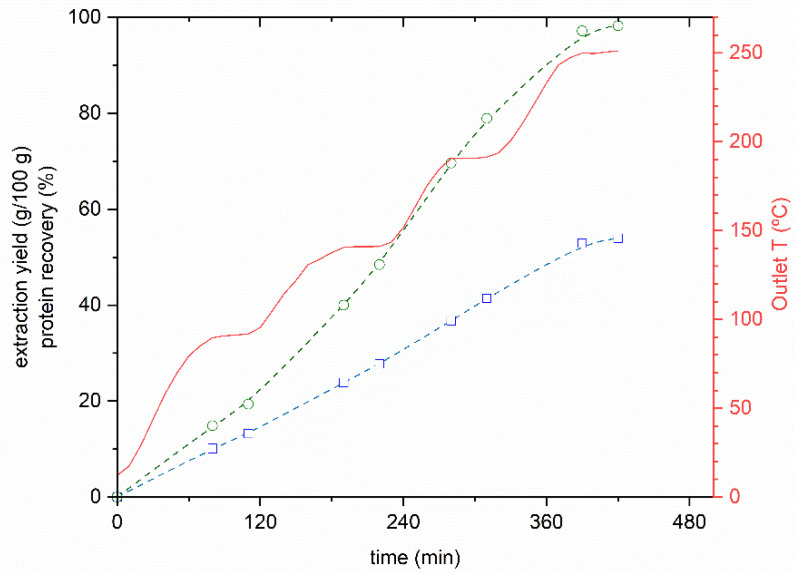
Temperature profile (solid line), extraction yield (□) and protein recovery (○) during subcritical water hydrolysis of codfish frames at target temperatures of 90, 140, 190, and 250 °C. Dotted lines are guidelines connecting data points.

SBW extracts obtained at target temperatures of 90, 140, 190 and 250 °C were lyophilized to calculate the extraction yield during the SBW experiments. The protein content of the samples was calculated from the respective nitrogen content and using the previous corrected NPCF of 5.1. The results obtained are shown in Figure 1.

From this figure, we can see that the extraction yield reached 53.9 g/100 g, with a nearly total protein recovery. The mode of operation with several heating ramps allows to collect samples at different target temperatures, giving an idea of the extraction yield and the extract characteristics at each temperature in order to further explore specific extraction conditions. An additional SBW run was tested using a single heating ramp from ambient temperature to 250 °C, and then maintaining this temperature constant for a further 50 min. This method was faster and resulted in a higher extraction yield (57.7 g/100 g) in 4 h of assay with a protein recovery of almost 100%.

Table 2 summarizes the results obtained at each of the target temperatures and with a single heating ramp. ca. 95% of the initial mass of codfish frames processed by SBW was recovered in the end of the assays, either as extract or final residue. The undetected material may be organic compounds decomposed to volatile species due to the high temperature conditions involved.

Regarding the composition of the SBW extracts, and as expected, protein was the major compound in all the SBW extracts, followed by mineral compounds. Lipid and carbohydrate compounds were found in minor quantities, closing the mass balance.

The residue left in the reactor after the SBW assays was almost completely composed of mineralized compounds, with protein present in traces or not detected, such as carbohydrates and lipids. These results demonstrate the deproteinization ability of the SBW method developed in this work and show its potential to produce pure HAp from codfish frames.

The amino acid profile of the SBW extracts obtained at different target temperatures was also analyzed by HPLC-IC method. First, extract samples were directly analyzed, omitting the alkaline digestion step to determine the presence of free amino acids. Then, the extracts were submitted to alkaline hydrolysis and subsequently analyzed for the total amino acid profile of protein fragments and peptides. As it can be observed from Table 3, free amino acids are present in low quantities at 90 °C, the lowest temperature studied in this work, accounting for approximately 2.6% of the total amino acids detected in the sample. When extraction temperature increases, the ratio of free to total amino acids increases up to 7.3 and 7.7% at 140 and 190 °C, respectively. Production of free amino acids reaches its maximum at 250 °C, the highest temperature studied in this work. At this temperature, 22% of the detected amino acids were in free form according to the analysis of the amino acid profile. Similar results have been reported in the literature, since temperatures in the range 220–260 °C have been reported as the optimal for amino acid production by SBW process, depending on the raw material and mode of operation [29,30,31,32,33,34,35,36,37,38,39,40]. In a previous work with sardine waste from fish canning, the highest yield of free amino acids was found at 140 °C and amino acid production decreased at higher temperatures due to thermal decomposition into organic acids and volatile carbon [40]. From these results, it is obvious that the amino acid profile and the nature of the proteins present in the raw material, together with the operating temperature and residence time (i.e., the severity of the SBW treatment) exerts an important influence in the production of free amino acids.

In relative terms, there are not many significant changes in the amino acid profiles of the SBW extracts obtained at the different temperatures studied in this work, and these profiles are also similar to that of the original codfish frames. These results indicate a low amino acid selectivity of the SBW process, which was already observed in a previous work with sardine waste [40]. The predominance of glycine, alanine and proline continues, although alanine was the most abundant amino acid instead of glycine at 90, 140 and 190 °C. At 250 °C, glycine increased again, likely due to amino acid transformation during SBW process [57].

### 3.3. Mineral Profile of Extracts

Major mineral compounds of extracts are shown in Table 4, together with the mineral profile of the raw material and the minerals found in the residue left after SBW process. Mineral recovery was calculated for each species, based on the mineral composition of the extracts and residue left after extraction, weighted by the extraction yield at each temperature and the final yield of mineralized residue. For each mineral compound, it can be observed that the calculated values are close to those obtained in the determination of the mineral profile of the raw material, validating the results obtained.

From Table 4, it can be seen that Ca, Na and P are the most abundant of the analyzed mineral compounds in the codfish extracts. Na is present in significantly higher quantities in the first extract, indicating that the more soluble sodium salts were extracted in the earlier stages of the SBW process at temperatures as low as 90 °C. On the other hand, Ca, P and Mg accumulated in the residue, meaning that hydroxyapatite and other mineralized structures were not significantly affected by SBW process up to 250 °C. Similar results were also found in a previous work dealing with SBW of sardine waste [40]. Heavy metals and toxic elements were absent or present in quantities within legal limits [58], which opens the utilization of SBW extracts from codfish frames in human food and pharmaceutical applications.

### 3.4. Molecular Weight Distribution of SBW Extracts

#### 3.4.1. SDS-PAGE of Codfish Extracts

**Figure 2:**
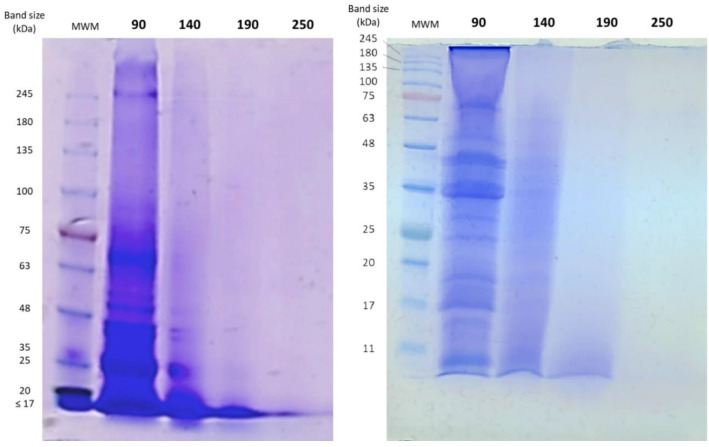
Polyacrylamide gels obtained in the SDS-PAGE of codfish SBW extracts at different temperatures (90, 140, 190, and 250 °C). **Left**: 7.5% precast acrylamide gel; **right**: handcast acrylamide gel (4.5% stacking and 15% resolving gel). MWM: molecular-weight marker.

Figure 2 shows the electrophoretic profiles of codfish extracts obtained after SBW process. The 7.5% precast gel depicted in the image on the left achieved better separation of the high-molecular weight bands. In the samples obtained at 90 °C, intense bands can be observed at high molecular weight (>245 kDa). These bands, which might correspond to non-hydrolyzed collagen components (β and γ chains), are less visible at 140 °C and not present at 190 and 250 °C, indicating the effectiveness of temperature in the hydrolysis of collagen from codfish frames.

Comparing the SDS-PAGE results obtained in this work with the electrophoretic patterns of Type I and II collagens reported in the literature for other fish species [39,58,59,60], the characteristic double bands found at 125 and 110 kDa, corresponding to α1 and α2 chains, are noticeably absent even at the lowest temperature assayed (90 °C). Instead, several bands that might correspond to degradation products of these α chains can be observed below 63 kDa (Figure 2, right). Again, these bands become less intense at higher hydrolysis temperatures. The absence of these bands in the hydrolysates has been reported in the literature for collagen hydrolysates obtained by enzymatic and other alternative methods, including SBW [39,59,60,61]. In the case of the extracts obtained at 190 and 250 °C, low molecular weight bands were observed near the bottom of the gel indicating the presence of the low mass proteins in the hydrolysate. The molecular weight of these bands was graphically determined and estimated to be less than 6 kDa, which is also in concordance with results from SBW of fish proteins available in the literature [35,60,61].

#### 3.4.2. SEC-GPC of SBW Extracts

The molecular weight distributions of the SBW extracts are shown as SEC-GPC curves in Supplementary Figure S1. The retention times of the molecular mass standards were 5.48 min for bovine Thyroglobulin (670 kDa), 6.82 min for γ-Globulin from bovine blood (150 kDa), 8.53 min for Albumin for chicken egg grade VI (44.3 kDa), and 9.70 min for Ribonuclease A type I-A from bovine pancreas (13.7 kDa). The data collected from the SEC-GPC chromatograms is presented in Table 5.

The SEC-GPC chromatogram of the SBW extract obtained at 90 °C showed several peaks at 4.05, 5.92, 7.95, and 9.65 min (Figure S1a), corresponding to high and medium molecular-weight peptides (estimated M_w_ of 1451, 361, 82.3, and 20.8 kDa, respectively) that might have been partially hydrolyzed and extracted in the subcritical water treatment at mild temperatures. Three more peaks are present at 11.20, 11.73 and 12.90 min, with estimated M_w_ of 5.6, 2.6 and 1.1 kDa, respectively. The highest peak observed in the SBW extract obtained at 90 °C presented a retention time of 12.92 min (1.1 kDa).

In the SBW extract obtained at 140 °C (Figure S1b), the first peak is observed at RT = 9.35 min (87.7 kDa), indicating that most of the high molecular-weight compounds at this relatively higher temperature have been hydrolyzed into smaller peptides. The highest peak was observed at 11.25 min (5.7 kDa), and a lower peak was also observed at 12.9 min (0.9 kDa). In the SBW extracts obtained at 190 and 250 °C (Figure S1c,d), the highest intensity peaks were observed at 11.3 min (3.2 to 5.9 kDa); thus, the position of the highest peak in the SEC-GPC curves shifted to a longer retention time with increasing process temperature in SBW. Several small peaks were also observed at RT > 15 min, likely corresponding to degradation products with M_w_ < 1 kDa.

As a general trend, the SEC-GPC chromatograms showed that high and medium molecular-weight peptides (20–1500 kDa) were extracted at the lowest temperature (90 °C). Increasing SBW temperature up to 140 °C promoted the hydrolysis of larger proteins and peptides, and low molecular-weight products (M_w_ around 4–6 kDa) appeared in the extract. Further temperature increments up to 190 and 250 °C enhanced the hydrolysis process, and only small molecules with M_w_ < 4 kDa were obtained, some of them being smaller than 1 kDa. Similar results have been reported by other authors. Koomyart et al. [62] treated semidried Isada krill with subcritical water at 100–240 °C interval and used SEC chromatography to qualitatively analyze the molecular mass distribution of their extracts. They observed that SBW extracts collected at 100–140 °C had several peaks corresponding to molecular masses higher than 1000 kDa, while at temperatures higher than 160 °C, a greater number of lower-molecular mass compounds (<10 kDa) were detected. Ueno et al. [37] when degrading fish gelatin with subcritical water at temperatures between 160 and 240 °C have reported an increase in the number of the peptides formed with increasing the temperature, by SEC-GPC analysis of the respective extracts. The retention time of the highest peaks observed for extracts obtained at 160–200 °C were ca. 4.5 min (303 kDa) and the retention times for the 220–240 °C extracts were ca. 5.0 min (235 kDa). Collagen isolated from Mackerel bone and skin, processed with SBW at 200–250 °C [39], resulted in the production of smaller molecular-weight compounds, ranging from 790 to 1400 Da, with the application of high temperatures.

### 3.5. Anti-Inflammatory Potential of Codfish SBW Extracts on Caco-2 Cells

Chronic inflammation is related to the development and progression of many chronic diseases such as autoimmune diseases, metabolic disorders, fibrosis, and cancer. Nutrition patterns influence the inflammation level, and any healthy diet should take into consideration the impact on systemic inflammation [63]. Thus, the obtained extracts were evaluated as potential anti-inflammatory food ingredients.

**Figure 3:**
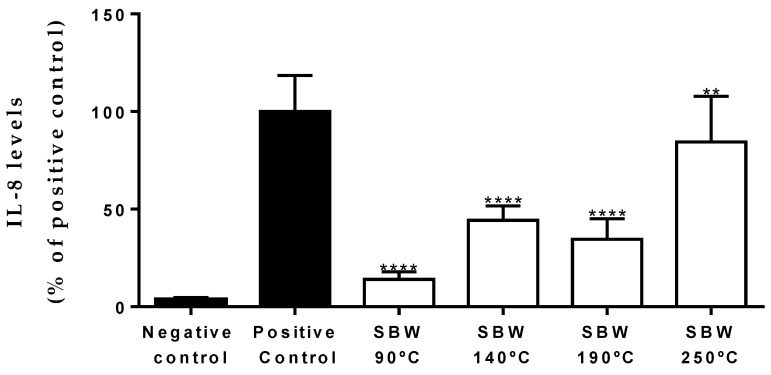
IL-8 produced by Caco-2 cells treated with cod fish SWH extracts at different temperatures (90, 140, 190, and 250 °C) and using a cocktail of cytokines (IL-1β, TNFα and LPS) to mimic inflammation. The results are expressed as mean % positive control ± SD, *n* = 5. ** *p* < 0.01, **** *p* < 0.0001, significantly different when compared to positive control, using *t*-test (*p* < 0.05).

Codfish SBW extracts were firstly tested for their cytotoxicity using confluent and undifferentiated Caco-2 cells as a model for crypt enterocytes [47]. Results obtained after 24 h of culture conclude that codfish SBW extracts did not show cytotoxicity to Caco-2 cell line in the range of tested concentrations (EC50 > 50 mg/mL). To determine the anti-inflammatory potential of codfish SBW extracts, Caco-2 cells were incubated with the extracts at 25 mg/mL and with a cocktail of cytokines (IL-1β, TNF-α and LPS). The expression of the interleukin-8 (IL-8), one of the major mediators of the inflammatory response, in cell culture supernatants was quantified through ELISA and is shown in Figure 3. According to Matias et al. [64], the secretion of IL8 is correlated to the modulation NF-κB pathway. When the transcription factor NF-κB is activated, it migrates to the nucleus and promotes the transcription of proinflammatory genes.

Results show that all codfish SBW extracts lead to a decrease in IL-8 release, comparing with positive control, which means that NF-κB pathway is inhibited. SBW 90 °C is the extract that shows higher decrease of inflammation, (14.1 ± 0.7%, a reduction of 85.9% comparing to the positive control; **** *p* < 0.0001). SBW 140°C and SBW 190°C exhibit an IL-8 reduction of 55.7% (44.3 ± 1.4; **** *p* < 0.0001 vs. positive control) SBW 65.5% (34.5 ± 2.1%; **** *p* < 0.0001 vs. positive control), respectively. On the other hand, SBW 250°C is the extract that presents the lowest anti-inflammatory potential, with a reduction of 15.5% in the release of IL-8 (84.5 ± 4.9%; ** *p* < 0.01 vs. positive control). These results are in accordance with previously published works regarding the anti-inflammatory potential of low molecular weight fish peptides [65,66], and suggest that anti-inflammatory peptides are produced and extracted at low temperatures; whereas at higher temperatures up to 250 °C, the degradation of these compounds into smaller peptides, free amino acids and other substances without anti-inflammatory activity occurs. A similar behavior has been observed in the production of antihypertensive peptides from fish gelatin using SBW [37].

### 3.6. Hydroxyapatite Isolation and Characterization

As described in Section 2, the deproteinized residue that remained after the SBW assay, together with the residues obtained after direct heating or alkaline digestion of the codfish frames, were analyzed by FT-IR spectroscopy, X-ray diffraction, and FEG-SEM microscopy.

#### 3.6.1. FT-IR Characterization

**Figure 4:**
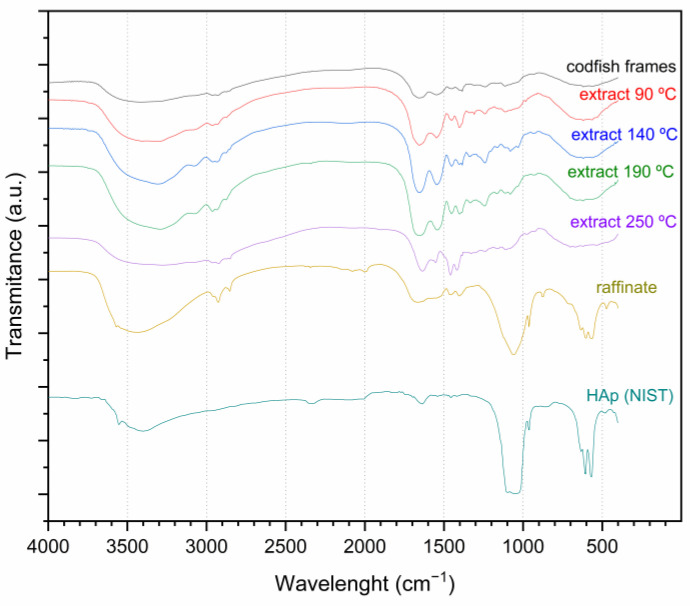
FT-IR spectra of powdered codfish frames, extracts obtained from subcritical water hydrolysis at different temperatures and the residues left after SBW process, thermal decomposition at 550 °C, and alkaline hydrolysis of codfish frames. Spectra of calcium phosphate hydroxide taken from NIST database [48].

FT-IR spectroscopy provides information about the vibrational behavior of inter and intramolecular bonds; thus, it can be used for structural investigation of the extracellular matrix of codfish frames. Figure 4 shows the FT-IR spectra of untreated codfish frames, SBW extracts obtained at different temperatures, and the residues obtained after SBW process, direct heating at 550 °C, and alkaline extraction by 4.2 M NaOH solution at 110 °C. The spectrum of pure calcium phosphate hydroxide obtained from the NIST database [48] is also shown for comparison. From this figure, important differences can be observed between the spectra of the raw codfish frames and the residues from SBW, direct heating, and alkaline extraction, revealing that the bone chemical structure has significantly changed during treatment. Spectra of the residues are similar to that of the pure HAp standard, most notoriously for the SBW residue. These results suggest that the residue obtained after SBW process is mostly composed of highly pure HAp.

Characteristic bands of HAp, related to OH stretching (at 3569 cm^−1^) and liberation (635 cm^−1^), as well as intense phosphate contours (from 500 to 1100 cm^−1^) can be observed in all the residues and pure HAp standard. These bands are not so clear in the original codfish frames due to the cross-linked structure of codfish frames and the presence of collagen proteins that limited vibrational behavior.

The SBW extracts obtained at different temperatures were also submitted to FT-IR spectroscopy since this technique may provide useful information on the protein structure through the analysis of the three main amide regions: I, II, and III. Amide I bands have been reported at 1622, 1636 and 1685 cm^−1^ in collagen/hydroxyapatite composites [67]. As it can be seen in Figure 4, amide I bands appear at these wavelengths in the original codfish frames as well as in the SBW extracts. According to Chang and Tanaka [67], Amide II major band appears at 1559 cm^−1^, and some minor bands at 1521, 1533 and 1543 cm^−1^. Figure 4 shows Amide II peaks in the original codfish frames as well as in the SBW extracts. Characteristic peaks of Amide III band appear at 1231, 1248, and 1281 cm^−1^ [67] and are typically of low intensity. In codfish frames, we observed some small peaks displaced to 1235, 1269, and 1295 cm^−1^. A similar displacement of the Amide III region was also observed by Barakat et al. [41] and was attributed to changes in the degree of cross-linking between the natural bone and the collagen/hydroxyapatite composite used in that study. Comparing the FT-IR spectra of the SBW extracts with that of the original cod fish frames, we can see that the chemical structure of the proteins was not substantially modified after SBW process. Peaks of the Amide I, II and III regions appear with higher intensity in the SBW extracts, which is likely due to the higher protein purity in the extracts and to the loss of the cross-linked structure of the bone that limited the vibration of the functional groups of the peptide linkage. Other noticeable peaks that can be taken into consideration appeared at 1398 and 1452 cm^−1^. These new peaks present higher intensity at 250 °C, contrary to the peaks from Amide I and II regions, which gain in intensity from 90 to 190 °C and then decrease at 250 °C. Since Amide I and II bands are related to the stretching and bending of the functional groups of the peptide linkage, their lower intensity at 250 °C suggest that protein hydrolysis is widely extended, as it has been confirmed by the high concentration of free amino acids at this temperature, as well as the results from SDS-PAGE and SEC-GPC analysis.

From Figure 4, it can be observed that both the Amide I, II and III bands and the new peaks observed in the SBW extracts are absent in the spectra of the codfish residues, confirming the ability of the SBW treatment for the protein removal and its equivalence to conventional HAp production methods such as thermal decomposition and alkaline digestion. Moreover, the similarity of the FT-IR spectrum of the SBW residue with that of the HAp standard suggest a high purity and crystallinity of the obtained HAp, which were further investigated by X-ray diffraction.

#### 3.6.2. X-ray Diffraction Profiles

X-ray diffraction (XRD) was considered to evaluate the presence of crystalline HAp in the residue left after SBW process. The residues of thermal decomposition at 550 °C and alkaline hydrolysis of codfish frames were also submitted to XRD analysis. The obtained XRD spectra were compared with NIST data from HAp standard [49].

**Figure 5:**
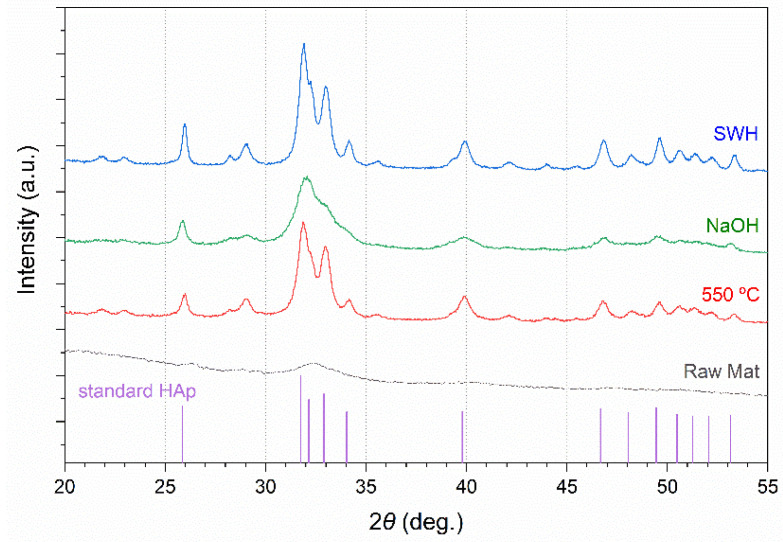
X-ray diffraction patterns of powdered codfish frames, and the residues left after SBW process, thermal decomposition at 550 °C, and alkaline hydrolysis of codfish frames. Standard HAp data taken from NIST SRM 2910b [49].

As shown in Figure 5, the spectrum of raw codfish frames only shows a small, diffuse peak around the 2*θ* = 32° region. Most likely, X-ray radiations have been dispersed by the fibrous collagen present in the bone extracellular matrix. On the other hand, the spectra of the samples obtained after thermal calcination and the SBW process are almost identical and show all the peaks corresponding to the standard HAp. Noteworthy, peaks were sharper in the spectra of the SBW treated codfish frames, suggesting that the SBW process can produce HAp with comparable or even higher crystallinity than the conventional thermal decomposition method. In the case of the spectrum of residue from alkaline hydrolysis, peaks were less defined and some of them could not be accurately identified, revealing that crystallinity and purity of HAp in this sample was not as high as in the others.

Table 6 shows the planar spacings (estimated by Bragg’s law) and the intensities at the strongest peaks in the XRD spectra. These results were compared with standard HAp data from NIST database and the relative errors were estimated at every plane. As it can be concluded from the total error values, the HAp obtained by the subcritical water process has planar spacings and intensities very close to the standard HAp, which indicates high crystallinity. Next is the product of thermal decomposition of codfish frames (550 °C, 4 h), followed by the residue obtained after alkaline digestion (4.2 M NaOH 110 °C, 24 h). Similar results were obtained by Barakat et al. [41] since their alkaline hydrothermal method also produced less crystalline HAp, compared to SBW and thermal decomposition processes.

#### 3.6.3. FEG-SEM and EDS Characterization

**Figure 6:**
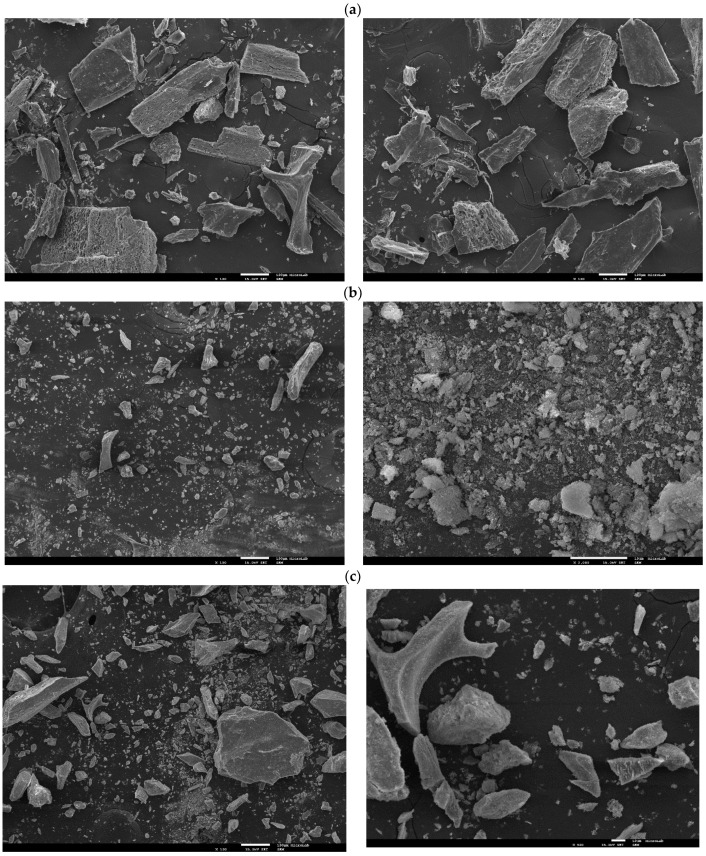

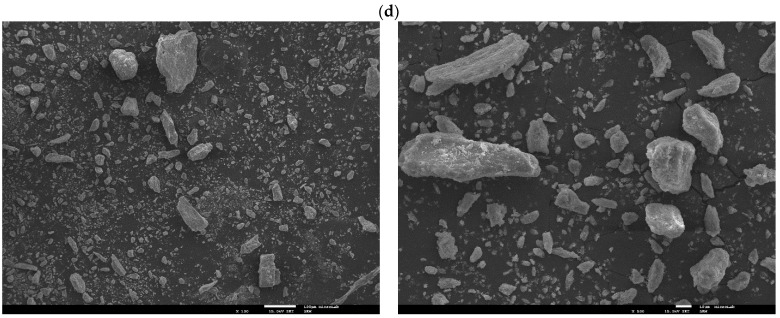
FEG-SEM micrographs of (**a**) grinded codfish frames (100× and 500×); (**b**) codfish frames after subcritical water treatment (100× and 2000×); (**c**) codfish frames after thermal decomposition at 550 °C (100× and 500×); (**d**) codfish frames after alkaline hydrolysis (100× and 500×).

The morphology of the residue obtained after SBW process was checked by FEG-SEM. The grinded raw material and the residues from the muffle treatment at 550 °C and the NaOH hydrolysis were also analyzed for comparison. The obtained micrographs are shown in Figure 6.

As shown in Figure 6a, the grinded raw bone has a solid surface with porous regions that might correspond to the internal surfaces of fractured bones. The morphology of some of the particles still preserves characteristics of the original codfish frames and some fibrillar structures, possibly from the adhered muscle, can be observed.

The effect of the SBW process can be observed in Figure 6b. In this case, the original morphology of the codfish frames is not present. Some large, irregular bone fragments can be observed but the SBW residue mostly consists of small particles agglomerated in clusters with sizes ranging from 0.1 to 10 µm. This morphology was also observed by Barakat et al. [41]. Subcritical water was able to dissolve the protein and other organic compounds nearly completely in the bone matrix, leaving the hydroxyapatite powder observed in Figure 6b.

For the residues of the thermal decomposition at 550 °C and the alkaline hydrolysis processes, the obtained morphologies are shown in Figure 6c,d, respectively. From Figure 6c, it seems that the thermal decomposition process preserves the morphology of the original codfish frames, since large and irregular particles have been obtained. No fibrillar structures or porous surfaces were observed, indicating that organic matter is removed with this treatment. Contrary to the results obtained in this work, Barakat et al. obtained HAp particles with nanorod shape and average size of 300 nm, although they used higher temperatures for the thermal decomposition (750 °C) which likely promoted the crystallization process [41]. On the other hand, the alkaline hydrolysis has similar effect to that of the SBW process. As shown in Figure 6b,d, both processes produced small hydroxyapatite flakes although larger particles were observed in the case of the alkaline hydrolysis process.

Complementarily to the FEG-SEM analysis, energy-dispersive X-ray analysis was performed in different surface areas of the obtained HAp powders. Results are shown in Supplementary Figures S2–S5. According to the chemical formula of the standard hydroxyapatite, the calcium to phosphorous molar ratio is approximately 1.67. The EDS analysis gave average Ca/P ratios of 1.62 ± 0.21, 1.90 ± 0.5, and 1.51 ± 0.1 for the residues obtained by SBW process, thermal decomposition at 550 °C, and alkaline hydrolysis, respectively. All the obtained values lie within the acceptable range for hydroxyapatite [56]. In the case of the raw codfish frames, Ca and P and other minerals commonly present in HAp were not detected in the surface of the raw codfish frames, likely because the protein matrix dispersed the X-ray radiation. The eventual presence of elements Na and Cl in the residues is interesting to check since their presence or absence may be related to the different treatment methods. Na and Cl were not detected in the SBW residue since they were solubilized and removed by the subcritical water treatment. However, Na and Cl were present in the residue from the thermal decomposition at 550 °C, whereas for the residue from the alkaline hydrolysis the element Cl was absent and Na residues from the NaOH treatment were detected.

## 4. Conclusions

The results presented in this study demonstrate the feasibility of the integral valorization of codfish (*Gadus morhua*) frames. Using green extraction methods such as subcritical water extraction and hydrolysis (SBW), codfish frames were fractionated into their main constituents, namely proteins and minerals, each of them with promising applications in the food, pharmaceutical, biomedical, and cosmetic industries. Proteins in codfish frames were almost entirely recovered as aqueous extracts, obtaining peptides of decreasing molecular weight when increasing the extraction temperature from 90 to 250 °C. The protein extracts were non-cytotoxic in a model for crypt enterocytes (Caco-2 cells), yet they showed high anti-inflammatory potential in human intestinal epithelium cell mode. The main constituent of the mineral fraction, hydroxyapatite, was isolated in an almost pure and crystalline form; its potential applications are currently under study. In view of these results, it is clear that the proposed SBW process promotes a more effective use of the waste generated during codfish processing.

## Figures and Tables

**Table 1:** Proximate composition, amino acid profile and main minerals found in codfish frames.

Proximate Composition		Amino Acid Profile			Mineral Profile	
(g/100 g)		Amino Acid	(mg/g)	(% rel.)	(mg/g)	
Moisture	5.4 ± 0.1	Arginine (Arg)	37.67 ± 0.1	8.1	Ca	143.0
Protein	47.0 ± 0.9 *	Lysine (Lys)	12.98 ± 0.1	2.8	Na	5.3
Lipids		Alanine (Ala)	58.52 ± 1.8	12.6	P	69.4
Total	1.9 ± 0.1	Threonine (Thr)	16.00 ± 0.5	3.4	Mg	2.43
Neutral	1.4 ± 0.1	Glycine (Gly)	75.52 ± 0.7	16.2	Zn	0.0351
Ash	39.3 ± 1.1	Valine (Val)	19.66 ± 0.1	4.2	Fe	0.0073
Carbohydrates	0.3 ± 0.05	Serine (Ser)	21.96 ± 0.2	4.7	Cr	n.d.
		Proline (Pro)	55.60 ± 1.9	12.0	As	0.0004
Collagen content	30.6 ± 0.8	Hydroxyproline (Hyp)	40.99 ± 1.0	8.8	Cd	n.d.
		Leucine (Leu)	18.58 ± 0.6	4.0	Hg	0.0001
		Isoleucine (Ile)	16.66 ± 0.3	3.6	Pb	0.0003
		Methionine (Met)	12.85 ± 0.2	2.8		
		Histidine (His)	11.68 ± 0.05	2.5		
		Phenylalanine (Phe)	11.64 ± 0.04	2.5		
		Glutamic acid (Glu)	19.40 ± 0.4	4.2		
		Aspartic acid (Asp)	17.86 ± 0.1	3.8		
		Cysteine (Cys)	1.28 ± 0.1	0.3		
		Tyrosine (Tyr)	16.25 ± 0.3	3.5		
		Total	465.1 ± 8.5	100.0		

n.d.: not detected; * Determined by elemental analysis with a specific NPCF of 5.1 ± 0.3.

**Table 2:** Extraction yield and composition of extracts and final residue obtained from subcritical water hydrolysis of codfish frames.

Temperature (°C)	Extraction Yield (g/100 g)	Protein Content (wt %)	Ash Content (wt %)
Mode of operation: heating ramps (90, 140, 190 and 250 °C)			
25–90	13.2	81.6	15.4
90–140	27.7	93.6	2.7
140–190	41.4	95.0	2.5
190–250	53.9	84.4	13.2
Residue		0.1	99.6
Mode of operation: single heating step (up to 250 °C)			
25–250	57.7	84.4	10.1
Residue		n.d.	99.8

Experimental uncertainty (u): u (extraction yield) = ±0.5 g/100 g; u (protein content) = ±0.3 wt %; u (ash content) = ±0.3 wt %

**Table 3:** Amino acid profile of extracts obtained from subcritical water hydrolysis of codfish frames.

	Amino Acid Profile (mg/g)							
	90 °C		140 °C		190 °C		250 °C	
	Free	Total	Free	Total	Free	Total	Free	Total
Arginine (Arg)	5.22	47.14	16.67	56.85	14.06	39.74	27.83	54.75
Lysine (Lys)	0.19	35.01	3.77	36.50	1.15	32.53	4.91	24.54
Alanine (Ala)	5.40	155.56	3.31	135.56	3.07	132.41	30.67	111.41
Threonine (Thr)	n.d.	4.13	2.52	4.56	1.00	9.97	n.d.	24.42
Glycine (Gly)	1.39	116.66	3.31	107.28	6.30	118.49	33.35	145.04
Valine (Val)	0.32	10.30	2.45	15.64	2.26	10.24	7.02	3.17
Serine (Ser)	n.d.	44.93	0.40	52.48	0.54	76.51	n.d.	81.08
Proline (Pro)	1.75	90.20	3.95	92.88	6.09	116.63	9.71	88.11
Hydroxyproline (Hyp)	1.59	88.91	3.55	81.61	5.56	86.12	12.07	100.27
Leucine (Leu)	0.18	23.78	0.89	18.66	0.60	4.29	6.53	24.52
Isoleucine (Ile)	0.42	20.52	2.76	30.82	3.55	59.83	14.61	37.74
Methionine (Met)	0.29	6.05	2.84	5.60	0.68	24.20	4.13	7.77
Histidine (His)	0.09	8.44	4.62	4.25	0.62	5.22	1.40	4.39
Phenylalanine (Phe)	0.62	5.02	4.25	18.79	3.07	4.05	3.82	3.15
Glutamic acid (Glu)	0.40	30.46	n.d.	98.33	n.d.	1.10	0.00	22.69
Aspartic acid (Asp)	0.32	26.63	1.86	18.39	4.45	5.31	4.45	12.67
Cysteine (Cys)	n.d.	n.d.	n.d.	0.86	n.d.	0.70	n.d.	0.98
Tyrosine (Tyr)	0.49	5.26	3.09	42.77	3.51	6.31	5.16	8.70
Total	18.67	719.00	60.24	821.83	56.50	733.65	165.67	755.40
Free/total ratio	0.026		0.073		0.077		0.22	

Experimental uncertainty (u) = ±0.1 mg/g extract; n.d.: not detected.

**Table 4:** Major minerals and toxic and heavy metals found in extracts obtained from subcritical water hydrolysis of codfish frames.

Mineral	Codfish Frames	Extract 90 °C	Extract 140 °C	Extract 190 °C	Extract 250 °C	Residue after SBW	Recovery mg/g Feed
Major mineral compounds (mg/g)							
Ca	143	2.58	0.38	0.27	0.51	410	147.2
Na	5.3	17.70	0.78	0.82	0.59	2.1	3.9
P	69.4	1.54	1.76	1.56	0.45	221	80.5
Mg	2.43	0.40	0.12	1.10	0.21	8.5	3.7
Zn	0.0351	0.061	0.008	0.052	0.025	0.03	0.057
Fe	0.0073	0.005	0.001	0.003	0.002	0.008	0.006
Toxic and heavy metals (ppm)							
Cr	n.d.	n.d.	n.d.	n.d.	n.d.	n.d.	
As	0.4	0.09	0.09	0.28	n.d.	0.79	0.440
Cd	n.d.	n.d.	n.d.	n.d.	n.d.	n.d.	
Hg	0.1	n.d.	n.d.	n.d.	n.d.	n.d.	
Pb	0.3	0.28	0.28	0.21	0.1	0.4	0.409

Experimental uncertainty (u) = ±5%; n.d.: not detected.

**Table 5:** Results obtained from the SEC-GPC chromatograms of the SBW extracts.

Peak No.	Max. RT (min)	M_p_ (kDa)	M_n_ (kDa)	M_w_ (kDa)	PD *	Area %
Codfish extract at 90 °C						
2	5.92	331.7	329.7	360.9	1.0946	3.21
3	7.95	62.5	67.4	82.3	1.2220	6.32
4	9.65	15.5	18.8	20.8	1.1078	7.54
5	11.2	4.33	5.09	5.61	1.1010	25.68
6	11.73	2.80	2.49	2.57	1.0293	17.14
7	12.90	1.07	0.96	1.08	1.1305	38.99
Codfish extract at 140 °C						
1	9.35	19.8	36.1	87.7	2.4287	36.36
2	11.25	4.16	4.08	5.69	1.3941	52.38
3	12.92	1.06	0.77	0.92	1.1961	11.04
Codfish extract at 190 °C						
1	11.3	3.99	0.98	5.93	6.0706	96.12
Codfish extract at 250 °C						
1	11.28	4.05	2.20	3.21	1.4588	65.59
2	14.02	0.43	0.16	0.30	1.8868	23.55

* Polydispersity, PD = M_w_/M_n._

**Table 6:** Planar spacings and intensities obtained from X-ray diffraction of the residue left after SBW process, thermal decomposition at 550 °C, and alkaline hydrolysis of codfish frames. Results are compared with standard HAp data from NIST SRM 2910b [63].

*h k l*	d-Value (nm)				Intensity			
	NIST	Muffle 550 °C	NaOH 110 °C	SBW 250 °C	NIST	Muffle 550 °C	NaOH 110 °C	SBW 250 °C
0 0 2	0.3444	0.3429	0.3445	0.3429	34	28	46.7	44
2 1 0	0.3084	0.3076	n.d.	0.3079	15	28	n.d.	25
2 1 1	0.2815	0.2808	0.2796	0.2805	100	100	100.0	100
1 1 2	0.278	0.2714	n.d.	0.2776	48	76	n.d.	75
3 0 0	0.272	n.d.	n.d.	0.2714	60	n.d.	n.d.	73
2 0 2	0.2632	0.2627	n.d.	0.2633	21	26	n.d.	27
3 1 0	0.2263	0.2259	n.d.	0.2264	22	33	n.d.	30
2 2 2	0.1944	0.1940	0.2261	0.1940	28	26	30.1	28
2 1 3	0.1842	0.1837	n.d.	0.1837	30	27	n.d.	27
3 2 1	0.1807	0.1802	0.1938	0.1804	15	21	26.9	21
0 0 4	0.1722	0.1719	0.1812	0.1717	13	16	20.5	20
Total Error		0.0459	n.c.	0.0230		4.50	n.c.	3.43

n.d.: not detected, n.c.: not calculated.

## Data Availability

Not applicable.

## Supplementary Materials

The following are available online at https://www.mdpi.com/article/10.3390/foods10061222/s1, Figure S1: SEC-GPC chromatograms of the extracts obtained by subcritical water hydrolysis of codfish frames at (a) 90 °C, (b) 140 °C, (c) 190 °C, and (d) 250 °C, Figure S2: Energy-dispersive X-ray analysis of the grinded codfish frames, Figure S3: Energy-dispersive X-ray analysis of the codfish frames after subcritical water treatment, Figure S4: Energy-dispersive X-ray analysis of the codfish frames after thermal decomposition at 550 °C, Figure S5: Energy-dispersive X-ray analysis of the codfish frames after alkaline hydrolysis.
