# Supplementary material for: Subcritical Water Extraction and Hydrolysis of Cod (Gadus morhua) Frames to Produce Bioactive Protein Extracts
Source: Foods. 2021 May 28;10(6):1222. doi: 10.3390/foods10061222 (PMC8228765; doi:10.3390/foods10061222)
Supplement: Supplementary file 1 [file foods-10-01222-s001.zip › foods-1227616-supplementary.pdf]

Supplementary Materials

Subcritical Water Extraction and Hydrolysis of Cod (*Gadus morhua*) Frames to Produce Bioactive Protein Extracts

Rodrigo Melgosa [1,*], Marta Marques [1], Alexandre Paiva [1], Ana Bernardo [2], Naiara Fernández [2], Isabel Sá-Nogueira [3] and Pedro Simões [1,*]

[1] LAQV-REQUIMTE—Associated Laboratory for Green Chemistry (LAQV) of the Network of Chemistry and Technology (REQUIMTE), Departamento de Química, Faculdade de Ciências e Tecnologia, Universidade Nova de Lisboa, 2829-516 Caparica, Portugal; r.melgosa@fct.unl.pt (R.M.); martadiasmarques@gmail.com (M.M.); abp08838@fct.unl.pt (A.P.)

[2] IBET-Instituto de Biologia Experimental e Tecnológica, Food & Health Division, 2781-901 Oeiras, Portugal; ana.bernardo@ibet.pt (A.B.); naiara.fernandez@ibet.pt (N.F.)

[3] UCIBio-REQUIMTE, Applied Molecular Biosciences Unit (UCIBIO) of the Network of Chemistry and Technology (REQUIMTE), Departamento de Ciências da Vida, Faculdade de Ciências e Tecnologia, Universidade Nova de Lisboa, 2829-516 Caparica, Portugal; isn@fct.unl.pt

* Correspondence: pcs@fct.unl.pt; Tel.: +351-212-948-385

List of Contents in Supplementary Materials

Figure S1. SEC-GPC chromatograms of the extracts obtained by subcritical water hydrolysis of codfish frames at a) 90 °C, b) 140 °C, c) 190 °C, and d) 250 °C

Figure S2. Energy-dispersive X-ray analysis of the grinded codfish frames

Figure S3. Energy-dispersive X-ray analysis of the codfish frames after subcritical water treatment

Figure S4. Energy-dispersive X-ray analysis of the codfish frames after thermal decomposition at 550 °C

Figure S5. Energy-dispersive X-ray analysis of the codfish frames after alkaline hydrolysis

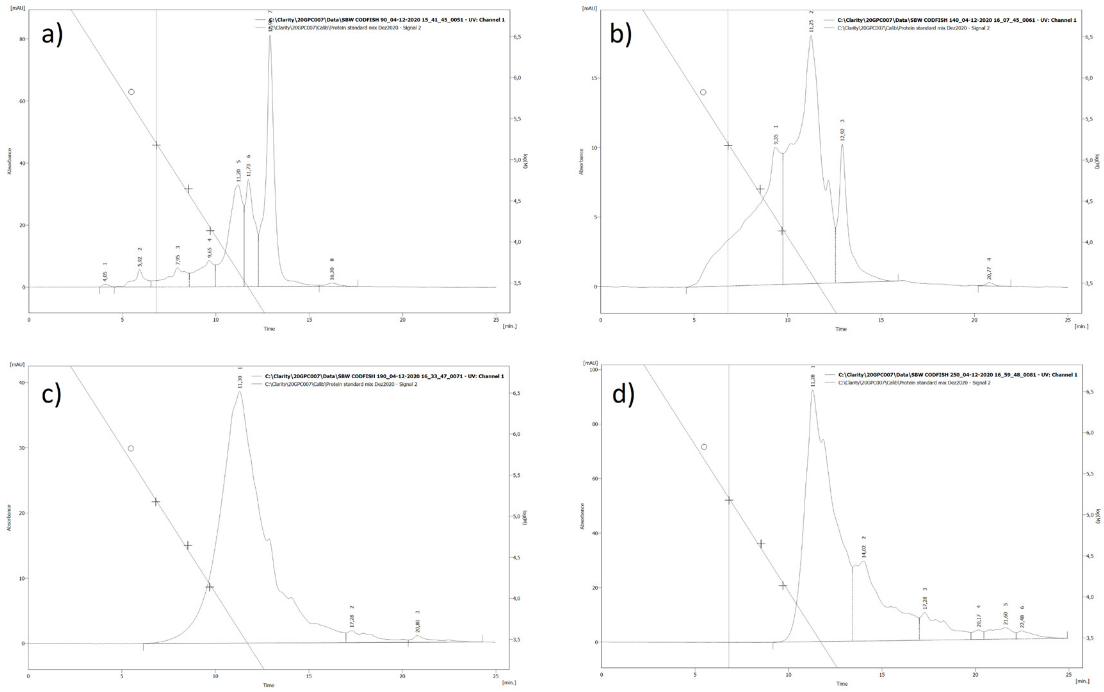

Figure S1. SEC-GPC chromatograms of the extracts obtained by subcritical water hydrolysis of codfish frames at a) 90 °C, b) 140 °C, c) 190 °C, and d) 250 °C

Element	App Conc.	Intensity Corrn.	Weight%	Weight% Sigma	Atomic%
C K	14.05	1.1539	62.68	1.51	77.56
O K	2.68	0.6299	22.01	1.17	20.44
S K	0.29	0.9195	1.62	0.29	0.75
K K	0.14	1.0112	0.71	0.20	0.27
Au M	1.91	0.7588	12.98	1.00	0.98
Totals			100.00		

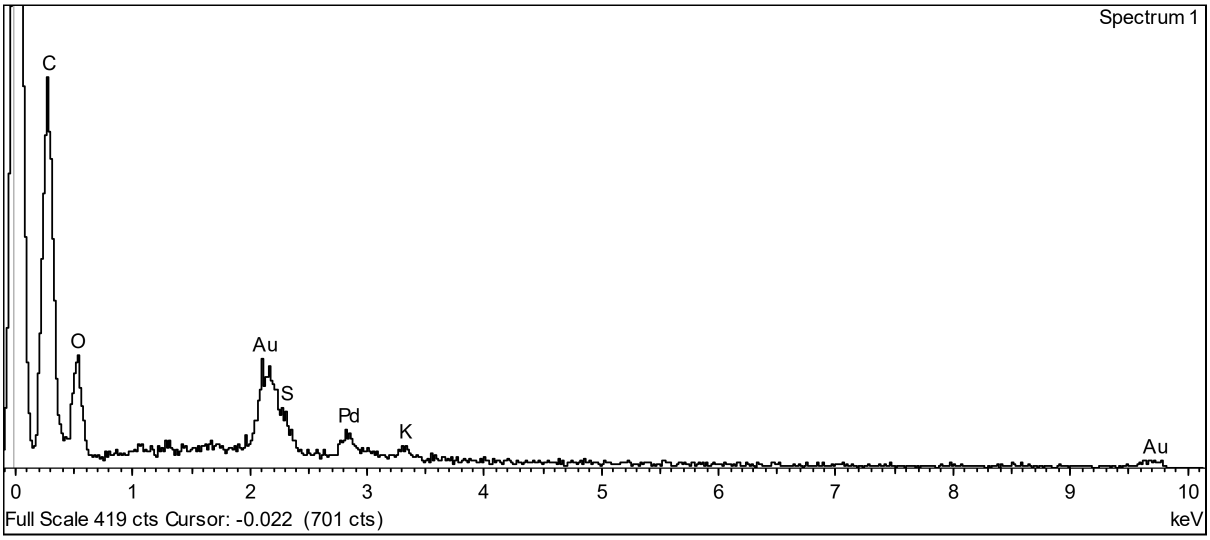

Figure S2. Energy-dispersive X-ray analysis of the grinded codfish frames

Element	App Conc.	Intensity Corrn.	Weight%	Weight% Sigma	Atomic%
C K	0.89	0.5096	4.66	0.71	10.42
O K	5.63	0.5290	28.47	0.77	47.81
Mg K	0.16	0.9280	0.47	0.10	0.52
P K	9.54	1.4906	17.09	0.39	14.82
Ca K	14.09	1.0193	36.91	0.63	24.74
Au M	3.70	0.7973	12.40	0.77	1.69
Totals			100.00		

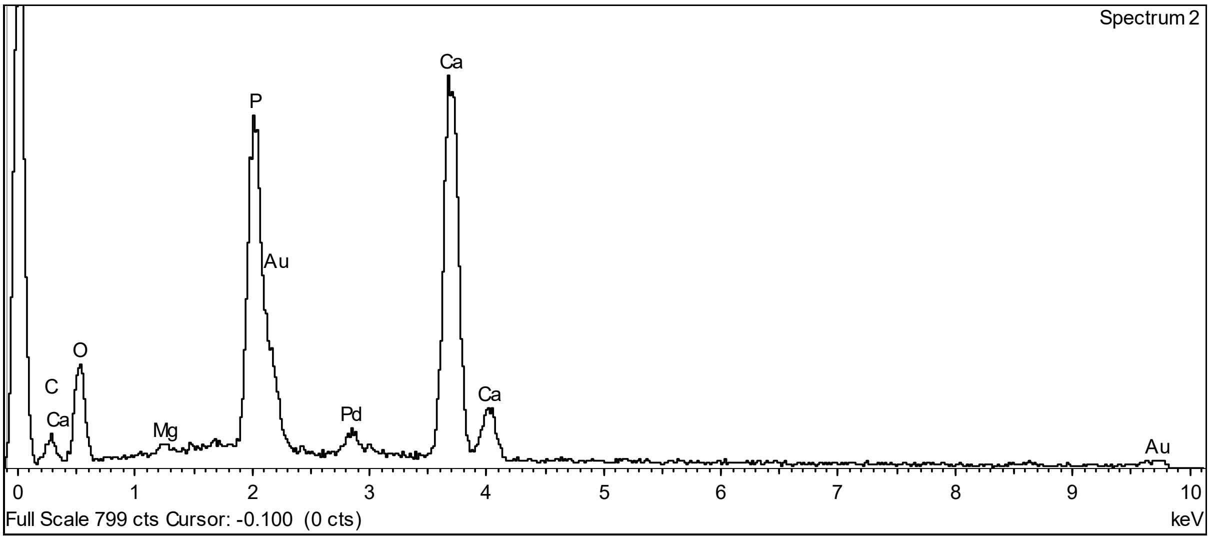

Figure S3. Energy-dispersive X-ray analysis of the codfish frames after subcritical water treatment

Element	App Conc.	Intensity Corrn.	Weight%	Weight% Sigma	Atomic%
O K	13.37	0.5866	45.71	0.58	65.39
Na K	0.48	0.9785	0.98	0.14	0.98
Mg K	0.23	0.8643	0.53	0.11	0.50
P K	12.50	1.4288	17.55	0.31	12.97
Cl K	0.21	0.8301	0.50	0.12	0.32
K K	0.45	1.1193	0.80	0.12	0.47
Ca K	17.07	1.0092	33.92	0.43	19.37
Totals			100.00		

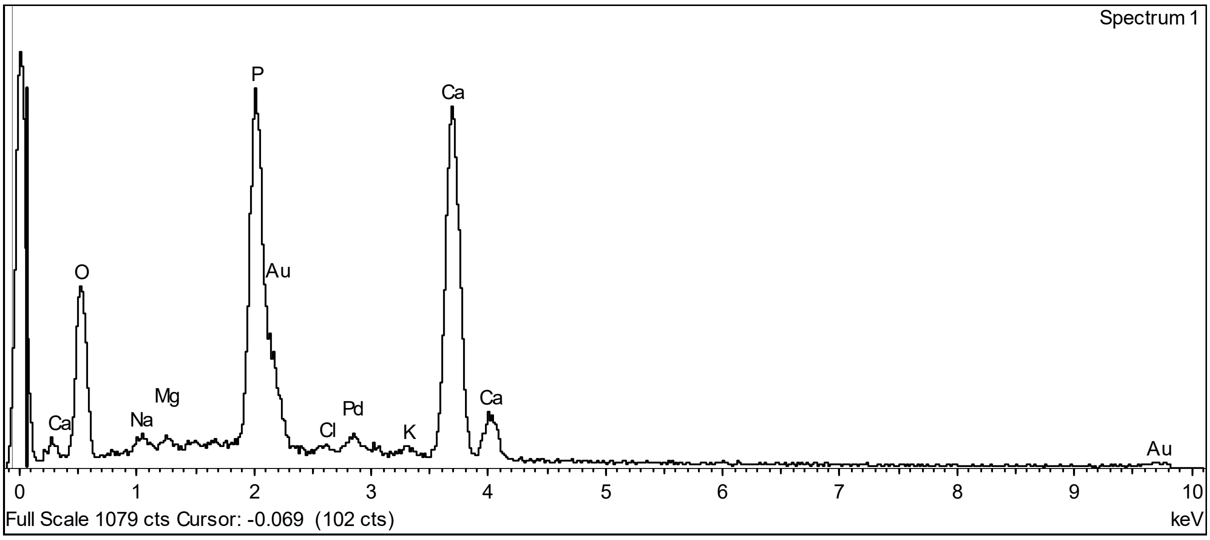

Figure S4. Energy-dispersive X-ray analysis of the codfish frames after thermal decomposition at 550 °C

Element	App Conc.	Intensity Corrn.	Weight%	Weight% Sigma	Atomic%
C K	1.26	0.5182	5.07	0.63	10.44
O K	10.11	0.5997	35.31	0.67	54.61
Na K	0.65	1.0493	1.30	0.13	1.40
Mg K	0.18	0.9077	0.42	0.10	0.42
Si K	0.43	1.0601	0.85	0.10	0.75
P K	10.54	1.4581	15.13	0.33	12.09
Ca K	14.75	1.0105	30.53	0.48	18.85
Au M	4.25	0.7814	11.39	0.65	1.43
Totals			100.00		

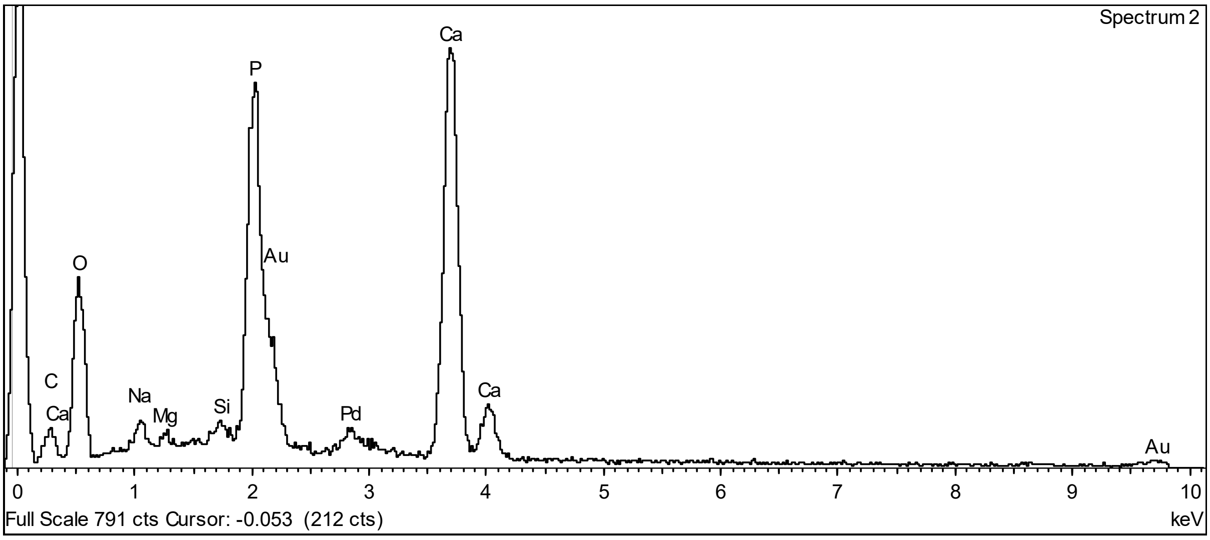

Figure S5. Energy-dispersive X-ray analysis of the codfish frames after alkaline hydrolysis
